# Clinical validation of the terminological subset for people with chronic kidney disease undergoing conservative treatment

**DOI:** 10.1590/1980-220X-REEUSP-2023-0280en

**Published:** 2024-02-12

**Authors:** Harlon França de Menezes, Alessandra Conceição Leite Funchal Camacho, Paola Paiva Monteiro, Isabele Silva dos Santos, Ana Beatriz Pereira, Nanete Caroline da Costa Prado, José Rebberty Rodrigo Holanda, Richardson Augusto Rosendo da Silva

**Affiliations:** 1 Universidade Federal Fluminense, Escola de Enfermagem Aurora de Afonso Costa, Programa Acadêmico em Ciências do Cuidado em Saúde, Niterói, RJ, Brazil.; 2 Universidade Federal Fluminense, Escola de Enfermagem Aurora de Afonso Costa, Departamento de Fundamentos de Enfermagem e Administração, Niterói, RJ, Brazil.; 3 Universidade Federal Fluminense, Escola de Enfermagem Aurora de Afonso Costa, Niterói, RJ, Brazil.; 4 Universidade Federal do Rio Grande do Norte, Departamento de Enfermagem, Programa de Pós-Graduação, Natal, RN, Brazil.; 5 Universidade Federal do Rio Grande do Norte, Programa de Pós-Graduação em Enfermagem, Natal, RN, Brazil.; 6 Universidade Federal do Rio Grande do Norte, Escola Multicampi de Ciências Médicas, Caicó, RN, Brazil.

**Keywords:** Conservative Treatment, Clinical Nursing Research, Validation Study, Renal Insufficiency, Chronic, Standardized Nursing Terminology, Tratamiento Conservador, Investigación en Enfermería Clínica, Estudio de Validación, Insuficiencia Renal Crónica, Terminología Normalizada de Enfermería, Tratamento Conservador, Pesquisa em Enfermagem Clínica, Estudo de Validação, Insuficiência Renal Crônica, Terminologia Padronizada em Enfermagem

## Abstract

**Objective:**

To clinically validate a terminological subset of the International Classification for Nursing Practice (ICNP^®^) to care for people with chronic kidney disease undergoing conservative treatment.

**Method:**

Prospective study of clinical validation assessment of 117 nursing diagnoses/outcomes statements and 199 nursing intervention statements. It was operationalized through the following steps: implementation of the Nursing Process in an outpatient clinic in Southeast Brazil; preparation of case studies; analysis of agreement between specialist nurses. The Kappa. Kruskal-Wallis coefficient of agreement and intraclass correlation coefficient (ICC) were used.

**Results:**

The sample consisted of 50 people with chronic kidney disease. Diagnoses/outcomes and interventions were evaluated with almost perfect/perfect agreement and excellent ICC. The Kruskal-Wallis test showed that there was no significant difference between the assessments. The study allowed the clinical validation of a subset with 110 nursing diagnoses/outcomes and 195 nursing interventions.

**Conclusion:**

Care for people with chronic kidney disease undergoing conservative treatment based on the proposed subset has become applicable to clinical practice.

## RESUMO

**Objetivo:**

Validar clinicamente um subconjunto terminológico da Classificação Internacional para a Prática de Enfermagem (CIPE^®^) para o cuidado às pessoas com doença renal crônica em tratamento conservador.

**Método:**

Estudo prospectivo. de avaliação de validação clínica de 117 enunciados de diagnósticos/resultados e 199 enunciados intervenções de enfermagem. Foi operacionalizado pelas seguintes etapas: implementação do Processo de Enfermagem em um ambulatório no Sudeste do Brasil; elaboração dos estudos de casos; análise de concordância entre enfermeiros especialistas. Utilizou-se o coeficiente de concordância de Kappa. Kruskal-Wallis e coeficiente de correlação intraclasse (CCI).

**Resultados:**

A amostra foi constituída por 50 pessoas com doença renal crônica. Os diagnósticos/resultados e intervenções foram avaliadas com concordância quase perfeita/perfeita e CCI excelente. O teste de Kruskal-Wallis mostrou inexistir diferença significativa entre as avaliações. O estudo possibilitou validar clinicamente um subconjunto com 110 diagnósticos/resultados e 195 intervenções de enfermagem.

**Conclusão:**

O cuidado às pessoas com doença renal crônica em tratamento conservador pautado no subconjunto proposto se tornou aplicável à prática clínica.

## RESUMEN

**Objetivo:**

Validar clínicamente un subconjunto terminológico de la Clasificación Internacional para Práctica de Enfermería (CIPE^®^) para la atención de personas con enfermedad renal crónica sometidas a tratamiento conservador.

**Método:**

Estudio prospectivo que evaluó la validación clínica de 117 declaraciones de diagnóstico/resultado y 199 declaraciones de intervención de enfermería. Fue operacionalizado a través de los siguientes pasos: implementación del Proceso de Enfermería en un ambulatorio del Sudeste de Brasil; preparación de estudios de caso; Análisis de concordancia entre enfermeros especialistas. Se utilizaron los coeficientes de concordancia Kappa. Kruskal-Wallis y coeficiente de correlación intraclase (CCI).

**Resultados:**

La muestra estuvo compuesta por 50 personas con enfermedad renal crónica. Los diagnósticos/resultados e intervenciones se evaluaron con una concordancia casi perfecta/perfecta y un CCI excelente. La prueba de Kruskal-Wallis mostró que no había diferencias significativas entre las valoraciones. El estudio permitió validar clínicamente un subconjunto con 110 diagnósticos/resultados y 195 intervenciones de enfermería.

**Conclusión:**

La atención a personas con enfermedad renal crónica sometidas a tratamiento conservador basado en el subconjunto propuesto se ha vuelto aplicable a la práctica clínica.

## INTRODUCTION

Chronic kidney disease (CKD) has emerged as a substantial issue for public health, being associated with high morbidity and mortality. The estimated global prevalence of CKD is 8–16%, with an increasing mortality rate, accounting for 1.2 million deaths. The global prevalence and mortality of CKD has increased dramatically since 1990, driven by an aging population and an increase in people with diabetes and hypertension which along with glomerulonephritis, are the leading causes of CKD^(1–3)^.

The burden of disability from a disease is an important factor that reflects the severity of an illness on a scale from 0 (perfect health) to 1 (equivalent to death), and represents the sum of years of life lost and those lived with disability. With regard to CKD, a rate of 35 million disability-adjusted life years is estimated^(2,3)^.

As it is a chronic condition that requires the involvement of a multidisciplinary team, care practices must be implemented in order to contribute to reducing the progression of the disease. In this way, conservative treatment is instituted in order to provide the person with CKD with a good and reconcilable clinical condition, especially with regard to treatment possibilities compatible with the reality of each individual, with a view to endorsing, at an opportune moment, a better condition, to discuss and decide on the most appropriate and convenient therapeutic method for future phases^(4)^.

Therefore, nurses gain prominence as they are inserted in this context with the objective of developing prevention and maintenance in a systematic way, based on instruments that contribute to comprehensive care, that is, that understand the person in their biological, emotional, and social context^(5)^.

A powerful technological instrument that can support nurses in providing standardized care, and which is based on the indication of phenomena relevant to their practice, are the terminological subsets of the International Classification for Nursing Practice (ICNP^®^). These bring together indications of diagnosis/result statements (ND/NO) and nursing interventions (NI), which are aimed at specific groups of people, and use the ICNP^®^ terms and structure. Therefore, it is possible to characterize it as a specialized language that is representative of local and global nursing practice, and that promotes the appropriate use of the nursing process, expressing itself in a strategy of competent collection, storage, and analysis of nursing data, resulting in the recognition of the profession^(6)^.

Among the main existing nursing theoretical frameworks, the Roy Adaptation Model (RAM) stands out because it demonstrates that the individual or group interacts and responds to stimuli from the environment, making its suitability plausible for people with CKD who need to get adapted to their new health condition^(7,8)^.

Therefore, the quality of these instruments shall be checked to have their validity attested as a necessary step towards their legitimacy and credibility for care practice^(9)^. Clinical validation aims to confirm whether the components of proposed statements, such as titles, definitions, and magnitudes, developed and validated by experts, are supported by real clinical data from a specific population, and to apply tests that demonstrate statistical associations and represent the level of empirical validity of the instrument^(10)^.

Given the importance that validation studies have as they consist of a test of the use of the data collection instrument in clinical practice, thus verifying its suitability and showing the relevance of this research, this study aimed to clinically validate a terminological subset of the ICNP^®^ for the care of people with chronic kidney disease undergoing conservative treatment.

## METHOD

### Study Design Period and Local

Prospective and clinical validation study, which used case studies operationalized by the stages of the nursing process, carried out in a kidney disease outpatient clinic, located in a city in southeastern Brazil. This study adhered to the guide Standards for Quality Improvement Reporting Excellence (SQUIRE) from Enhancing the QUAlity and Transparency Of health Research (EQUATOR).

A terminological subset previously validated in terms of content was used as an empirical basis, consisting of 117 ND/NO and 199 NI statements, and categorized according to the adaptive modes proposed by Roy’s theoretical model (physiological, self-concept, interdependence, and role function). Such statements were constructed based on an integrative literature review, survey of terms, cross-mapping, construction of statements, and content validation with Brazilian specialist nurses^(11)^.

It is worth noting that the International Council of Nurses (ICN) announced a partnership with the Systematized Nomenclature of Medicine International (SNOMED International) to integrate ICNP® into SNOMED – Clinical Terms (SNOMED CT). Thus, in order to update the statements that made up the subset, a manual cross-mapping was carried out with SNOMED CT, where support was obtained from a sworn translator in the English language hired by the authors with the aim of equating the nomenclature concepts with the subset on screen^(12)^.

Of the diagnostic statements, 74 were equivalent with SNOMED-CT, with 41 allocated to the physiological mode. 17 to the self-concept mode, nine to the role function mode and six to the interdependence mode. The EI statements were prepared according to the aforementioned empirical basis.

### Population Selection Criteria and Sample Definition

The population for clinical validation consisted of people with CKD undergoing conservative treatment who were admitted to the program through a nursing consultation. To calculate the sample, the formula for finite populations was used, taking into account the 95% confidence level (Z∞ = 1.96), sampling error of 10%, population of 100, resulting in a sample of 50 people, who were selected consecutively for convenience.

The sample was defined based on the inclusion criteria: people with CKD, over 18 years of age and who were admitted for conservative treatment at the service during the data collection period. As exclusion criteria, those who did not have the cognitive capacity to participate in the research verified through the evaluation obtained through medical records and those who did not attend some of the scheduled appointments. Thus, based on these criteria, a sample of 50 participants was reached.

Also part of the sample were four specialist nurses (SN1, SN2, SN3, SN4) working in the service who met the following inclusion criteria: specializing in nephrology and who had clinical care experience of at least five years in the area. As an exclusion criterion, those on leave or vacation. It should be noted that there is a consensus in the literature that there is no minimum or maximum number of nurses to carry out data collection therefore, this value was determined for convenience^(13)^.

These nurses participated in remote training, which lasted five hours, carried out by the main researcher and a doctoral student for one week, to discuss the clinical inference process and the review of relevant topics related to data collection, people undergoing conservative treatment. On this same occasion, the terminological subset was presented, in order to reduce any biases that could harm the inferential process and determine the established criteria. Participants received the Free and Informed Consent Form.

### Data Collection

To achieve the objective, the following phases were operationalized: 1) Implementation of the Nursing Process according to the stages: data collection, diagnosis, planning, implementation, and nursing assessment; 2) Preparation of case studies and ND/NO/NI statements; 3) Analysis of agreement among specialist nurses. These steps were carried out between February and December 2022. Participants were selected from February 1st to February 28th of the same year.

To guide data collection, a protocol was used which established that the participant was monitored at three moments: upon admission (1st consultation), 30 days after admission (2nd consultation) and 60 days after admission (3rd consultation). This time interval was adopted as a criterion for data collection, as it is routine for the service for patients to return within the stipulated period. It should be noted that the pair carried out 50 consultations in the three moments and from them they described the case studies and developed the nursing process.

In the first stage, data collection and physical examination occurred simultaneously by the pair (main researcher and doctoral student). An instrument designed for the study was used in accordance with RAM, consisting of the following parts: sociodemographic data; nursing history, diagnoses, planning, implementation and assessment. Furthermore, laboratory tests and vaccination records were evaluated. A pilot test was carried out, after approval by the research ethics committee, to verify the suitability of the instrument, and participants were not included in the study.

To determine the ND/NO statements, the clinical indicators described in the operational definitions of the statements of the ICNP^®^ terminological subset were used as a basis. These definitions were already in a Google Docs form. If a new situation was identified for which a statement had not yet been constructed, ICNP^®^, version 2019/2020, and the guidelines for constructing statements were used, that is, terms of the axes corresponding to each statement were obligatorily included. Likewise, the clinical findings identified during data collection were also considered a nursing diagnosis^(14)^.

The planning and implementation stages were carried out in accordance with the NO statements expected for the ND, established by the subset, and the proposed NI were implemented by the duo (main researcher and doctoral student), and those that depended on collaboration of the participants were passed on through guidelines and delivered manually through educational material, in order to guarantee the implementation and continuity of the actions. And finally, in the evaluation stage, new data collection and physical examination were carried out, focusing on the identified ND statements, which occurred in the second and third consultations. Data collection for this phase took place from March 1st to May 31st 2022.

In the second phase, the pair prepared the case studies as well as the ND/NO/NI statements, which were inserted into a Microsoft Office Excel^®^ spreadsheet and into tabs, with each tab corresponding to a patient. The statements were organized according to the adaptive modes. These spreadsheets were sent for validation to the four specialist nurses. This phase took place from June 1st to August 31st 2022.

The third and final phase was the analysis of agreement between the four specialist nurses, who judged the applicability of the ND/NO/NI prepared by the pair through case studies. Thus, the experts received spreadsheets created by the duo (main researcher and a doctoral student), each referring to a patient and containing all the terms and ND/NO/NI according to the adaptive modes, as well as additional information regarding data socioeconomic, clinical and observations pertinent to the process of diagnostic inference.

In this way, each expert judged separately whether the ND/NO/NI were present or absent in each of the spreadsheets sent. For this research, the ND/NO and NI identified in people with chronic kidney disease, described in the case studies and present in the terminological subset, were considered clinically validated. This phase took place from September 1st to December 31st.

### Data Analysis and Treatment

The agreement between the four specialist nurses was tested by obtaining the Kappa Coefficient (k), with agreement being considered: poor (between 0 and 0.19); relative (0.20 to 0.39); moderate (0.40 to 0.59); substantial (0.60 and 0.79); almost perfect (0.80 to 0.99); and perfect (equal to 1)^(15)^.

The Kruskal-Wallis non-parametric test was used to test whether there was a difference in means in the assessment between specialist nurses. The p value was adopted with a significance level of 0.05, so that if the p value ≤ 0.05, the difference between the means was considered statistically significant. The Intraclass Correlation Coefficient (ICC) was applied to verify the reliability and usability of the subset in other contexts, considering that, at ICC ≥ 0.75, reliability must be considered excellent; in the ICC between 0.40–0.75, there is satisfactory reliability; and considered poor when the ICC < 0.40^(16)^.

Data related to ND/NO were analyzed using simple descriptive statistics, with absolute and relative frequency. The data related to NI were presented together, according to the category of the theoretical model, due to the number of statements, and discussed by frequency. The data were organized in an electronic data spreadsheet and, after double typing, were exported to the Statistical Package for Social Science (SPSS) version 20.0 Windows. After coding and tabulation, the data were analyzed by reflective and statistical reading using the Kappa test using the Online Kappa Calculator^(17)^.

For this research, the ND/NO and NI identified in the patients, described in the case studies and present in the terminological subset, were considered clinically validated^(18)^.

### Ethical Aspects

The research respected volunteering, anonymity, and confidentiality in light of regulatory standards for research involving human beings in accordance with the Resolution 466/12, having been approved by the Research Ethics Committee of a federal university, with opinion number 3.798.213, approved in 2020. All participants signed the Free and Informed Consent Form.

## RESULTS

The sample consisted of 50 people with chronic kidney disease undergoing conservative treatment of both sexes, 45.5% women and 54.5% men. Regarding age, 88.6% were 60 years old or over and only 11.3% were under 60 years old. The prevalent level of education was unfinished secondary education 47.7%; as for marital status 66% were married; and for income 41% had an income equal to or greater than two minimum wages.

All participants had some comorbidity, the most frequent of which were: high blood pressure (93.2%), diabetes mellitus in 84% of individuals. Kidney disease staging was performed using the CKD-EPI equation, with 27.2% having CKD in stage II, 52.2% in stage III, and 13.6% in stage IV.

In the study, 105 (90%) of the ND/NO that were included in the original subset were implemented, with an average of 14 statements per case study (SD = 5.08) and a minimum of eight and a maximum of 23. Regarding the ND/NO statements, 12 contained in the original subset were not identified in any case study, which are: “Functional Dyspnea/Dyspnea Absent”; “Risk of Nutritional Condition Impaired/Nutritional Condition Positive”; “Nausea/Nausea Absent”; “Skin Protective Capacity Impaired/Skin Protective Capacity Effective”; “Hypervolemia/Fluid Balance (or Fluid Balance), within Normal Limits”. “Cardiac Output Impaired/Cardiac Output Effective”; “Tactile Perception Impaired/Tactile Perception Effective”; “Postural Vertigo (Dizziness)/Postural Vertigo (Dizziness) Absent”; “Ability to Preparing Food Impaired/Ability to Prepare Food Improved”; “Safety Role Impaired/Safety Role Improved”; “Lack of Access to Transportation/Access to Transportation Improved”; “Lack of Trust in the Interprofessional Team/Trust in the Interprofessional Team”. Therefore, they have not been clinically validated in the context of care for people with CKD undergoing conservative treatment.

Tables 1, 2, 3, 4 present the distribution of ND/NO identified according to the RAM modes. The researchers identified the need to include five ND/NO that were not included, three of which came from academic, unpublished work that validated ND/NO/NI for hemodialysis patients and one was freely prepared, namely: “Literacy in Health Impaired”; “Anemia Chronic”; “Lack of Knowledge about Hemodialysis”; “Quality of Life Risk Negative”; “Religious Link Impaired”.

**Table 1:** Agreement of specialist nurses (SN) in relation to the Physiological Mode nursing diagnosis statements expressed by the Kappa coefficient – Rio de Janeiro, RJ, Brazil, 2022.

Nursing diagnoses/results – physiological mode	SN 1	SN 2	SN 3	SN 4	Kruskal-Wallis
**Oxygenation**					
Cough/Cough Absent	0.99	0.96	1.00	1.00	0.083
**Nutrition**					
Lack of Appetite/Appetite Positive	0.97	0.96	0.98	1.00	0.085
Ability to Manage (Control) Dietary Regime Impaired/Ability to Manage (Control) Dietary Regime Improved	1.00	0.94	0.98	0.99	0.064
Food Intake Behavior Compulsive/Eating Behavior Improved	1.00	0.90	0.87	0.98	0.083
Lack of Knowledge about Dietary Regime/Knowledge about Improved Dietary Regime	1.00	0.95	0.97	0.99	0.094
Weight Impaired/Weight within Normal Limits	1.00	0.99	0.97	0.98	0.401
**Elimination**					
Low Liquid Output/Liquid Output Improved	0.99	1.00	1.00	1.00	0.121
Liquid Imbalance/Liquid Balance (or Water Balance) within Normal Limits	0.95	1.00	0.99	1.00	0.601
Liquid Retention/Liquid Volume Effective	0.97	0.96	0.98	0.99	0.203
Constipation/Gastrointestinal System Function Effective	0.93	0.99	0.98	1.00	0.202
Risk of Constipation/Gastrointestinal System Function Effective	0.97	0.92	1.00	0.99	0.105
Pain during Urination (or Dysuria) Frequent/Pain during Urination (or Dysuria) Absent	0.95	0.96	1.00	0.95	0.337
Urinary Frequency Decreased/Urinary Frequency Normal	1.00	0.92	0.91	0.95	0.401
Urinary Elimination Decreased/Urinary Elimination Improved	0.97	0.98	0.99	1.00	0.201
Proteinuria/Urinary System Function Effective	1.00	0.95	0.91	0.98	0.404
**Activity and Rest**					
Ability to Manage (Control) Physical Exercise Regime Impaired/Ability to Manage (Control) Physical Exercise Regime Improved	0.96	0.99	0.97	1.00	0.063
Hypoactivity/Tolerance to Activity Related to Pathological Process Effective	0.97	0.99	0.94	1.00	0.104
Fatigue/Fatigue Reduced	1.00	0.97	0.93	0.99	0.525
Leg Cramp/Leg Cramp Improved	0.99	0.98	0.94	1.00	0.502
Fall Risk/Fall Risk Control	0.96	0.98	0.93	1.00	0.401
Sleep Impaired/Sleep Adequate	0.94	0.99	0.99	1.00	0.071
Discomfort/Comfort Improved	0.99	0.95	0.98	1.00	0.413
**Protection**					
Skin Integrity Impaired/Skin Integrity Improved	0.98	0.95	1.00	0.99	0.102
Skin Dry/Skin Dry Improved	1.00	0.96	0.99	0.98	0.105
Skin Integrity Risk Impaired/Skin Integrity Risk Control	0.92	0.99	0.96	1.00	0.201
Itching/Itching Improved	0.99	0.98	0.97	1.00	0.062
Immunization Regimen Impaired/Immunization Regimen Improved	0.98	0.96	1.00	0.99	0.401
Teething Impaired/Teething Improved	0.98	0.95	1.00	0.99	0.092
Exposure to Contamination/Exposure to Contamination Decreased	0.90	1.00	0.92	0.95	0.123
Risk of Urinary Infection/Urinary Infection Risk Control	0.95	1.00	0.96	1.00	0.401
Susceptibility to Infection/Infection Control	0.99	0.98	1.00	1.00	0.201
Inflammation Chronic/Inflammation Chronic Improved	0.95	0.97	1.00	1.00	0.620
**Senses**					
Pain Acute/Pain Reduced	1.00	0.98	0.94	1.00	0.102
Pain Chronic/Pain Reduced	0.96	0.99	0.95	1.00	0.405
Musculoskeletal Pain/Musculoskeletal Pain Improved	1.00	0.98	1.00	0.99	0.101
Sensory Perception Impaired Auditory/Sensory Perception Enhanced	1.00	0.97	1.00	0.96	0.085
Sensory Perception Impaired Visual/Sensory Perception Enhanced	0.95	0.97	0.99	1.00	0.230
**Fluids and Electrolytes**					
Risk for Blood Pressure Change/Control of Blood Pressure	0.94	1.00	0.98	1.00	0.104
Risk of Arrhythmia/Arrhythmia Absent	0.98	1.00	0.93	0.97	0.301
Fluid Volume Impaired/Liquid Volume Effective	0.92	1.00	0.93	0.89	0.315
Anemia Chronic/Anemia Controlled	0.93	0.96	0.99	1.00	0.105
Peripheral Edema/Peripheral Edema Absent	0.92	0.95	0.98	1.00	0.052
Presence of Hyperphosphatemia/Electrolyte Balance Improved	0.91	0.97	0.99	1.00	0.120
Presence of Hypernatremia/Electrolyte Balance Improved	0.92	0.96	0.96	1.00	0.114
Presence of Hyperkalemia/Electrolyte Balance Improved	0.92	0.93	0.99	1.00	0.822
Presence of Hypocalcemia/Electrolyte Balance Improved	0.92	0.96	0.98	1.00	0.412
Presence of Hypophosphatemia/Electrolyte Balance Improved	0.96	0.99	0.98	1.00	0.134
Risk of Electrolyte Imbalance/Electrolyte Balance Improved	0.95	0.98	0.94	0.99	0.402
Metabolic Acidosis Present/Acid-Base Balance. Improved	0.98	1.00	0.98	1.00	0.423
Renal Function Impaired/Renal Function Effective	1.00	0.99	0.98	1.00	0.401
Cardiac Function Impaired/Cardiac Function Effective	1.00	0.97	0.99	1.00	0.061
Blood Pressure Altered/Blood Pressure within Normal Limits	1.00	0.96	0.99	1.00	0.082
Risk of Cardiac Function Impaired/Cardiac Function Effective	1.00	1.00	0.99	0.98	0.404
Risk of Liquid Volume Imbalance/Liquid Volume Control	1.00	1.00	0.99	0.97	0.231
**Neurological Function**					
Memory Impaired/Memory Effective	1.00	1.00	0.96	0.97	0.412
Learning Impaired/Learning Enhanced	1.00	0.95	0.97	0.98	0.402
Communication Impaired/Communication Effective	1.00	0.97	1.00	0.99	0.068
**Endocrine Function**					
Blood Glucose Self-Monitoring Impaired/Blood Glucose Self-Monitoring Improved	1.00	0.98	1.00	0.99	0.701
Hyperglycemia/Blood Glucose Level within Normal Limits	1.00	0.99	1.00	0.98	0.082
Hypoglycemia/Blood Glucose Level within Normal Limits	0.96	1.00	0.97	1.00	0.137
Risk for Blood Glucose Altered/Blood Glucose Level within Normal Limits	0.95	1.00	0.98	1.00	0.101
Hypovitaminosis Present/Hypovitaminosis Improved	0.99	1.00	0.90	1.00	0.148

**Table 2:** Agreement of specialist nurses (SN) in relation to the nursing diagnoses statements of the Self-Concept Mode expressed by the Kappa coefficient – Rio de Janeiro, RJ, Brazil, 2022.

Nursing diagnoses/outcomes – self-concept mode	SN 1	SN 2	SN 3	SN 4	Kruskal-Wallis
Self-image Negative/Self-image Positive	1.00	0.99	0.95	1.00	0.240
Stigma/Stigma Reduced	1.00	0.98	1.00	0.99	0.401
Sexual Performance Impaired/Sexual Performance Improved	1.00	0.99	1.00	0.98	0.074
Anxiety/Anxiety Reduced	0.98	0.99	1.00	1.00	0.071
Low Self-Esteem/Self-Esteem Positive	0.91	0.98	1.00	1.00	0.053
Spiritual Belief Conflicted/Spiritual Belief Enhanced	1.00	0.99	0.94	0.98	0.118
Adaptation Impaired/Adaptation Improved	0.99	1.00	0.98	1.00	0.101
Behavior Aggressive/Aggressive Behavior Absent	1.00	0.99	0.95	1.00	0.122
Hopelessness/Hope Improved	1.00	0.98	0.95	1.00	0.620
Difficulty Coping with the Illness/Difficulty Coping with the Illness Reduced	0.95	1.00	0.96	1.00	0.064
Mood Depressed/Depressed Mood Decreased	1.00	1.00	0.97	0.95	0.065
Lack of Resilience/Resilience Improved	0.94	1.00	0.96	1.00	0.241
Suffering/Suffering Reduced	0.95	1.00	1.00	0.99	0.071
Sadness/Sadness Reduced	1.00	1.00	0.99	0.97	0.095
Crying Present/Crying Absent	0.92	0.97	1.00	1.00	0.065
Spiritual Anguish/Spiritual Anguish Decreased	0.99	0.97	1.00	1.00	0.081
Fear of Death/Fear of Death Decreased	1.00	1.00	1.00	0.99	0.401
Fear of Abandonment/Fear of Abandonment Decreased	1.00	0.96	0.94	1.00	0.242
Fear of Being a Burden to Others/Fear of Being a Burden to Others Decreased	1.00	0.99	0.97	1.00	0.402

**Table 3:** Agreement of specialist nurses (SN) in relation to the nursing diagnoses statements of the Function in Real Life Mode expressed by the Kappa coefficient – Rio de Janeiro, RJ, Brazil, 2022.

Nursing diagnoses/outcomes – real life function mode	SN 1	SN 2	SN 3	SN 4	Kruskal-Wallis
Stress/Stress Decreased	1.00	0.98	0.96	1.00	0.148
Risk of Dissatisfaction with Health Care/Satisfaction with Health Care	0.99	1.00	1.00	0.98	0.223
Tobacco (or Smoking) Abuse/Tobacco (or Smoking) Abuse Absent	0.93	0.90	0.90	0.91	0.201
Ineffective Health Self-Control/Self-Control Improved (or Increased)	1.00	0.97	0.99	1.00	0.402
Self-Care Deficit/Able to Perform Self-Care	1.00	0.98	0.94	1.00	0.071
Ability to Manage (Control) the Medication Regimen Impaired/Ability to Manage (Control) the Medication Regimen Positive	1.00	0.97	0.98	1.00	0.140
Ability to Dress and Groom (Take Care of External Appearance) Impaired/Ability to Dress and Groom (Take Care of External Appearance) Improved	0.98	0.99	1.00	1.00	0.085
Acceptance of Health Status Impaired/Acceptance of Health Status Effective	0.98	1.00	0.97	1.00	0.071
Denial about the Severity of the Illness/Denial Absent	1.00	0.99	1.00	1.00	0.248
Expectation about Treatment Unrealistic/Expectation about Treatment Improved	1.00	0.98	1.00	1.00	0.219
Low Attendance at Follow-up Consultation (or Subsequent Consultation)/Attendance at Follow-up Consultation (or Subsequent Consultation) Improved	0.96	1.00	0.98	1.00	0.081
Continuity of Care Problem/Continuity of Care Effective	0.95	1.00	1.00	0.98	0.435
Role Performance Impaired/Role Performance Effective	1.00	0.95	1.00	0.99	0.701
Work role Impaired/Work Role Improved	1.00	0.97	1.00	0.98	0.345
Health Literacy Impaired/Health Literacy Improved	0.98	1.00	0.99	1.00	0.405
Religious Bond Impaired/Religious Bond Improved	1.00	0.98	0.95	1.00	0.305
Lack of Knowledge about Hemodialysis/Knowledge about Hemodialysis	1.00	0.93	0.97	1.00	0.418
Quality of Life Risk Negative/Quality of Life Risk Improved	1.00	0.96	0.99	1.00	0.401
Family Role Impaired/Family Role Improved	0.97	1.00	0.95	1.00	0.523

**Table 4:** Agreement of specialist nurses (SN) in relation to the nursing diagnosis statements of the Interdependence Mode expressed by the Kappa coefficient – Rio de Janeiro, RJ, Brazil, 2022.

Nursing diagnoses/outcomes – interdependence function mode	SN 1	SN 2	SN 3	SN 4	Kruskal-Wallis
Ability to Perform Leisure Activity Impaired/Ability to Perform Leisure Activity Improved	1.00	0.98	0.95	1.00	0.421
Access to Treatment Impaired/Access to Treatment Improved	1.00	0.95	1.00	0.97	0.257
Risk of Family Coping Harmed/Family Coping Effective	0.99	1.00	1.00	1.00	0.437
Family Decision-Making Process Impaired/Family Decision-Making Process Improved	1.00	0.96	1.00	1.00	0.253
Family Process Harmed/Family Process Effective	1.00	0.98	1.00	1.00	0.437
Communication between Interprofessional Team and Individual Impaired/Communication between Interprofessional Team and Individual Improved	1.00	0.95	0.99	1.00	0.420
Social Condition Impaired/Social Condition Improved	1.00	0.99	0.96	1.00	0.121
Lack of Social Support/Social Support Effective	0.92	1.00	0.95	1.00	0.440
Social Isolation/Social Isolation Decreased	0.91	0.99	1.00	1.00	0.436
Marital Satisfaction Impaired/Marital Satisfaction Improved	1.00	0.98	0.95	1.00	0.201

Regarding the IEs that were in the original subset 190 (96%) were implemented. The nursing interventions “Check for signs of low cardiac output”, “Evaluate laboratory tests”, “Monitor skin color”, “Promote good interaction”, “Remove common sense beliefs about the disease”, “Promote family communication”. “Evaluate role interaction”, “Evaluate dynamic functional relationship” and “Evaluate Genitourinary Condition”, contained in the original subset, were not identified in any case study, therefore, they were not clinically validated in the context of care for people with CKD undergoing conservative treatment. Furthermore, five new ones were added: “Implement strategies to understand the treatment”, “Identify the need for specialist medical evaluation”, “Promote knowledge about hemodialysis treatments”, “Evaluate quality of life”, “Know ways to return to religious ties”.

Table 5 presents the total number of nursing interventions identified in the participants followed in the case studies and the percentage in relation to the total number of interventions in the subset according to the adaptive modes.

**Table 5:** Nursing interventions identified in participants monitored in case studies. according to adaptive modes – Rio de Janeiro, RJ, Brazil, 2022.

Adaptive modes	N	(%)
Physiological Mode Oxygenation	03	100%
Physiological Mode Nutrition	09	81.8%
Physiological Mode Elimination	08	61.5%
Activity/Rest Physiological Mode	10	83.3%
Physiological Mode Protection	09	100%
Physiological Mode Senses	05	41.6%
Physiological Mode Fluids/Electrolytes	13	68.1%
Physiological Mode Neurological Function	09	90%
Physiological Mode Endocrine Function	11	100%
Self-Concept Mode	35	100%
Real-life Function Mode	24	82.4%
Interdependence Mode	31	83.7%

Diagnoses/outcomes and interventions were evaluated with near-perfect and perfect agreement, resulting in 110 diagnoses/outcomes and 195 nursing interventions. The Kruskal-Wallis test demonstrated that there was no statistically significant difference in the experts’ average assessments, demonstrating agreement between them. Finally, the global agreement between experts carried out using the intraclass correlation coefficient demonstrated the existence of excellent reliability in its entirety (ICC = 0.95), demonstrating the reliability and ability to use the subset in other contexts.

## DISCUSSION

As it is an asymptomatic disease, when CKD progresses to its advanced stage, often being detected late, it compromises control and the course of treatment. Thus, the recognition and monitoring of risk factors can contribute to the implementation of actions for comprehensive health promotion and disease prevention^(19)^. Factors such as motivation, information, socio-family support, and support from the healthcare team and services play a fundamental role in improving knowledge, coping and adaptation tools, and building individual self-confidence^(20)^.

Thus, the proposed terminological subset appears as a way of expressing care needs and the consequences that can be generated, allowing the institution of practical and shared actions. Consequently, from the moment the nurse makes use of this instrument, it allows their professional competence to be demonstrated through a reliable record that envisions the continuity of their care development.

To this end, the theoretical model adopted contributed to structuring the subset and guiding towards a nursing perspective with the adoption, in this case of adaptive modes. It should be noted that nursing theories represent structures for the development and testing of technologies and knowledge, as they represent in themselves care technologies relevant to the consolidation of good nursing and health practices^(21)^.

In the foreground, the Physiological Adaptive Mode covered a greater number of statements used by nurses since the subset brings together five major basic needs for physiological integrity, which meets the proposed theoretical model, and which contributed to the clinical assessment of people with chronic kidney disease. A study that evaluated the quality of life of people undergoing kidney treatment indicates that having one or more diseases simultaneously with CKD increases the burden of physical, psychological, emotional symptoms, and necessary care, which results in greater limitations, with consequent worsening of quality of life and disease progression^(22)^.

At the Physiological Adaptive Mode the statements of nursing diagnoses and outcomes portrayed specific responses from those who initiate treatment and which need to be adapted to a new condition arising from the illness, justifying the clinical validation of the proposed statements. Studies reveal that CKD causes uncomfortable body sensations and, consequently the lack of control of accepted risk factors for disease progression^(23,24)^.

Therefore, with the clinical assessments carried out, nurses act based on evidence founded on human responses and encouraging interventions that the individual can adopt^(25)^. This way, the application of care in a systematic way, as is the case with the use of the subset, allows the nurse’s judgment in understanding the indicators of their assessments that are most affected and which can direct planning that meets people’s specificities.

Concomitant to this aspect, comorbidities influence the progression of kidney disease on a broad spectrum. People undergoing conservative treatment participating in this study report having hypertension and diabetes as their main comorbidities. This data confirms that oxidative stress caused by hyperglycemia, as well as proteinuria, hyperperfusion, and renal hyperfiltration participate in the pathogenesis of chronic kidney disease. Furthermore, common factors associated with diabetes such as obesity and cardiovascular diseases, contribute greatly to the development of kidney damage^(26)^.

Faced with the magnitude of this problem in people’s lives, nurses have in their practice the establishment of clinical judgment about what physical responses cause, and in this way, implement nursing diagnoses to guide the nursing process. The use of standardized languages, as is the case with ICNP^®^, describes such knowledge and thus applies nursing interventions according to their particularities^(27)^. Such interventions are aimed at education, training, and behavioral change, which help people to gain more knowledge about conservative treatment and develop healthy lifestyle habits, further improving their adherence during this period.

An aspect that deserves to be highlighted was the statements of nursing diagnoses/results and interventions validated in psychosocial modes. It is known that a chronic disease causes numerous emotional consequences to a person’s life, and this aspect is often neglected in health practices. The statements were able to present satisfactory levels of agreement for everyday use.

An Australian study highlights the satisfaction of people with CKD regarding the role of nurses during the treatment process. On the other hand, the study points out that nurses should not focus only on the biological aspects of CKD related to lifestyle changes and medication adherence. Such professionals need to routinely assess psychosocial well-being to implement early and brief interventions that better support the emotional well-being of these people^(28)^. Furthermore, nursing interventions that apply educational, cognitive, behavioral, and dietary methods have been shown to exert favorable effects on the physical and emotional health of these people^(29,30)^.

The impact on the care practice of the subset in question is in the area of being a reference for outpatient care nurses. With this, an easy instrument is highlighted to guide these professionals in creating an individualized care plan. Institutions can establish subsets as a way of contributing to assistance by indicating statements and returning evidence, which can be transformed into clinical quality indicators. To this end, institutions need to gradually embrace management practices and encourage nurses to ensure knowledge, safety, and culture to minimize potential problems in these people.

Finally, we highlight that the evaluation of professionals when using the constructed subset was relevant. In this context, the evaluation occurred in a more homogeneous way, which indicates greater efficiency in the evaluation of patients, by providing scientific basis and validation of indicators capable of measuring real results and supporting the nursing care plan. As a limitation, the choice of a specific population may have limited the findings, making it impossible to generalize them. Furthermore, restricting the study to a single region and setting brings a simple cultural peculiarity.

## CONCLUSION

The study demonstrated the clinical validation of 110 ND/NO and 195 NI of the INCP^®^ terminological subset for people with chronic kidney disease undergoing conservative treatment. Clinical validation was identified for 90% of the ND/NO and 96% of the NI that make up the original subset. The statements showed almost perfect and perfect agreement and excellent overall agreement, demonstrating no statistically significant difference between the experts’ assessments. Thus, the construction of these empirical statements and the clinical validation process make nursing statements more suitable for specific populations and provide an effective means to better evaluate nursing actions.
